# Extension of the Shelf-Life of Fresh Pasta Using Chickpea Flour Fermented with Selected Lactic Acid Bacteria

**DOI:** 10.3390/microorganisms8091322

**Published:** 2020-08-30

**Authors:** Rosa Schettino, Erica Pontonio, Marco Gobbetti, Carlo Giuseppe Rizzello

**Affiliations:** 1 Department of Soil, Plant and Food Sciences, University of Bari, 70125 Bari, Italy; rosa.schettino@uniba.it (R.S.); erica.pontonio@uniba.it (E.P.); 2 Faculty of Science and Technology, Free University of Bozen-Bolzano, 39100 Bolzano, Italy; marco.gobbetti@unibz.it

**Keywords:** chickpea, lactic acid bacteria, sourdough, fresh pasta, antifungal activity, antifungal peptides

## Abstract

Fresh pasta is subjected to rapid spoilage, mainly due to the metabolic activity of bacteria, yeasts, and especially molds, which negatively affect the sensorial characteristics and the safety of the product. In this work, chickpea flour was fermented with selected lactic acid bacteria, characterized in terms of the antifungal activity, and used to fortify fresh semolina pasta. Pasta was characterized and subjected to a long period of storage after being artificially inoculated with *Penicillium roqueforti*. Conventional fresh semolina pasta, produced with or without calcium propionate addition, was used as a reference. The water/salt-soluble extract from chickpea sourdough exhibited antifungal activity towards a large spectrum of molds. Its purification led to the identification of ten potentially active peptides. Besides the high content of dietary fibers (4.37%) and proteins (11.20%), nutritional improvements, such as the decrease of the antinutritional factors concentration and the starch hydrolysis index (25% lower than the control) and the increase of the protein digestibility (36% higher than the control), were achieved in fresh pasta fortified with the chickpea sourdough. Inhibition of the indicator mold growth during a 40-day storage period was more effective than in pasta added to calcium propionate.

## 1. Introduction

Pasta, globally recognized as one of the most appreciated and accessible foods, is obtained by the extrusion or lamination of a dough made with semolina and water. A wide range of types of pasta, characterized by different shapes, compositions, and moisture contents, are produced worldwide [1]. The water content is the main factor affecting pasta’s shelf-life and storage conditions. Indeed, dried pasta (maximum 12.5% of water) can be stored at room temperature and has a shelf-life longer than one year. Conversely, fresh pasta (moisture between 24 and 30%) requires storage under refrigerated conditions and is easily perishable [1]. Nevertheless, the consumption of fresh pasta, either with or without fillings, is continuously increasing worldwide [2], with major requests in Asian (in which noodles and manti are very popular) and European countries [3]. The shorter cooking time compared to dried pasta (preferred in the catering sector) and the possibility to differentiate shapes and formulations (with other ingredients, such as vegetables, cheeses, fillings, and non-wheat flours) are the main factors affecting the choice of fresh rather than dried pasta.

Fresh pasta spoilage is mainly due to the metabolic activity of bacteria, yeasts, and especially molds [1], which negatively affect the sensorial characteristics and safety of the product [4,5,6,7]. Molds are derived from field (e.g., *Alternaria*, *Aureobasidium*, *Cladosporium*, *Epicoccum*, *Fusarium*, *Helminthosporium*, and *Claviceps*), post-harvest, or processing contamination (genera *Aspergillus*, *Penicillium*, and *Fusarium*) [8].

Overall, the industrial production of fresh pasta includes heat treatments (e.g., pasteurization) aimed at preserving product hygiene and quality [9]. However, other techniques can be applied, before and after packaging, such as microwave and convection heating, alone or in combination, in order to provide extensive and efficient sanitization, thus assuring shelf-life periods ranging from 30 to 90 days [10,11]. However, the thermal stress generally compromises the sensory characteristics of fresh pasta.

Indeed, the use of active compounds in the dough and modified headspace conditions during packaging [12,13] and gamma irradiation [14] have been proposed. Among the antimicrobial agents, potassium sorbate [15,16], sodium dehydroacetate, and calcium propionate, which are commonly used for fresh pasta preservation [17] have been proposed as suitable approaches for improving the microbial stability and extending the shelf life of fresh pasta. However, the consumer requirements of chemical-free products have forced researchers to move toward natural preservatives [18]. Indeed, “natural” antimicrobial compounds and food ingredients, such as chitosan [7], flaxseed flour [19], lemon extract and grapefruit seed extract [5], herb and spice extracts [20,21], and essential oils (including eugenol, thymol, menthol, and eucalyptol) [22], have already been investigated as fresh pasta additives.

In this framework, the use of microorganisms and/or their metabolites to prevent spoilage and extend the shelf life of foods is gaining interest as a bio-preservation approach. Lactic acid bacteria (LAB) are considered a valuable tool thanks to their ability to synthesize and release various antimicrobial and antifungal compounds from the matrix [23]. Synergistic activities between different compounds synthesized or released during fermentation, such as organic acids and peptides, can be responsible for the overall antifungal effect [23].

Although the conventional process for pasta does not include a fermentation step, novel recipes including LAB-fermented ingredients have recently been proposed, aimed at enhancing the nutritional and functional properties of this product and expanding the market supply with products exhibiting new sensorial profiles [24,25,26,27].

Here, chickpea flour was fermented with selected LAB and used to fortify fresh semolina pasta. The effect of the fortification on the microbiological shelf-life of the experimental pasta was investigated, and a mixture of peptides responsible for the antifungal activity were purified and identified. With the aim of assessing the effect of the fermented chickpea flour on the main features of the experimental pasta, an integrated characterization approach including technological, nutritional, and sensory investigations was also applied.

## 2. Materials and Methods

### 2.1. Raw Material, Bacterial Strains, and Fermentation

Chickpea grains (*Cicer arietinum* L. var. Pascià) provided by Caporal Grani S.a.s. (Gravina di Puglia, Italy) were used in this study. The whole grains were milled using a Ika-Werke M20 laboratory mill (GMBH, and Co. KG, Staufen, Germany). After milling, the flour was sieved (mesh size 500 nm) to remove the coarse fraction. The moisture, protein, lipids, total dietary fiber, and ash of raw material were determined according to Approved Methods 44–15A, 46–11A, 30–10.01, 32–05.01, and 08–01.01 of the American Association of Cereal Chemists [28]. Total nitrogen was corrected by 6.25 [29]. Carbohydrates were calculated as the difference [100 − (proteins + lipids + ash + total dietary fiber)]. Proteins, lipids, carbohydrates, total dietary fiber, and ash were expressed as % of dry matter (d.m.).

*Lactoplatibacillus plantarum* LB1 and *Furfurilactobacillus rossiae* LB5 [30] belonging to the Culture Collection of the Department of Soil, Plant and Food Science (University of Bari, Bari, Italy) and formerly known as *Lactobacillus plantarum* and *Lactobacillus rossiae* [31], respectively, were used as a mixed starter to ferment the chickpea flour. Strains were routinely cultivated on modified de Man Rogosa and Sharp (Oxoid, Basingstoke, Hampshire, UK) (mMRS), as reported by Rizzello et al. [30].

### 2.2. Chickpea Sourdough

A dough was prepared by mixing chickpea flour (62.5% w/w) with tap water (37.5%, w/w). The dough yield (DY, dough weight × 100/flour weight) was 160. To be used as a mixed starter for sourdough fermentation, LAB cells were harvested by centrifugation (10,000× *g*, 10 min, 4 °C) from the broth; washed twice in 50 mM phosphate buffer, pH 7.0; and re-suspended in tap water used for the dough making process (CS-T0, final cell density in the dough was ca. 7.0 log10 cfu/g). Fermentation was carried out at 30 °C for 24 h (CS-T24).

### 2.3. Chemical and Microbiological Characterization of Chickpea Sourdough

The pH value of dough and sourdough was determined online by a pH meter (Model 507, Crison, Milan, Italy) with a food penetration probe. The AACC method 02–31.01 [28] was used for determining the total titratable acidity (TTA) of samples.

Presumptive LAB were enumerated using de Man, Rogosa and Sharpe (MRS, Oxoid) agar medium, supplemented with cycloheximide (0.1 g/L). Plates were incubated, under anaerobiosis (AnaeroGen and AnaeroJar, Oxoid), at 30 °C for 48 h.

Water/salt-soluble extracts (WSE) from dough and sourdough were prepared according to the method originally described by Osborne [32] and modified by Weiss et al. [33]. WSE was used for organic acids, peptides, and total free amino acids (TFAA) analyses.

A High-Performance Liquid Chromatography (HPLC) ÄKTA Purifier system (GE Healthcare, Buckinghamshire, United Kingdom) equipped with an Aminex HPX-87H column (ion exclusion, Bio-Rad, Richmond, VA, USA) and a UV detector operating at 210 nm [34] was used to quantify organic acids. The fermentation quotient (FQ) was determined as the molar ratio between lactic and acetic acids.

TFAA were analyzed by a Biochrom 30 series Amino Acid Analyzer (Biochrom Ltd., Cambridge Science Park, United Kingdom) with an Li-cation-exchange column (20 by 0.46 cm internal diameter), as described by Rizzello et al. [34].

For the analysis of peptides, WSEs were treated with trifluoroacetic acid (TFA, 0.05% w/vol) and subjected to dialysis (cut-off 500 Da) to remove proteins and FAA, respectively. Then, the peptide concentration was determined by the *o*-phtaldialdehyde (OPA) method [35]. All analyses were carried out in triplicate. The supernatant was also used for the antifungal assay.

Raffinose and phytic acid concentrations were determined by using the Megazyme kit Raffinose/D-Galactose Assay Kit K-RAFGA and K-PHYT 05/07 (Megazyme International Ireland Limited, Bray, Ireland), respectively, following the manufacturer’s instructions. Condensed tannins were determined using the acid butanol assay, as described by Hagerman [36].

### 2.4. Fungi, Culture Media, and Growth Conditions

Penicillium polonicum CBS 112490, Penicillium chrysogenum CBS 111214, Penicillium paneum CBS 101032, Penicillium albocoremium CBS109582, Penicillium chermesinum CBS117279, Penicillium carneum CBS112297, Eurotium rubrum CBS150.92, Aspergillus parasiticus CBS971.97, Aspergillus versicolor CBS117286, Penicillium bialowiezense CBS110102, and Penicillium brevicompactum CBS28997 were obtained from the Culture Collection of Centraalbureau voor Schimmelcultures (Utrecht, Holland). Penicillium roqueforti DPPMAF1, Penicillium aethiopicum DPPMAF2, and Aspergillus niger DPPMAF3, acquired from the Culture Collection of the Department of Soil, Plant, and Food Sciences (Bari, Italy), were also used.

Fungi were grown in Potato Dextrose Agar (pH 5.6) (PDA, Oxoid Laboratories) at 25 °C for 24–72 h.

*P. roqueforti* DPPMAF1 was used as the indicator microorganism for antifungal assays since it corresponds to one of the most common cereal-derived spoilage molds, presenting a high resistance to the preservatives commonly added to baked goods [37,38].

### 2.5. Hyphal Radial Growth Rate Assay

The inhibitory activity of WSE was assayed based on the hyphal radial growth rate of fungi [39]. WSE was sterilized by filtration on 0.22 μm membrane filters (Millipore Corporation, Bedford, MA 01730, USA) and added (30%, *v/v*, final concentration) to sterilized PDA.

After mixing, aliquots of 20 mL were poured into Petri plates (90 mm diameter). Control plates contained PDA supplemented with 30% (*v/v*) of 50 mM sterilized Tris-HCl pH 8.8. The assay was carried out by placing a 3-mm diameter plug of growing mycelia into the center of Petri dishes containing the culture medium. Plates were incubated aerobically at 25 °C. Three replicates were run simultaneously. The radial growth of mycelia (colony diameter, mm) in all plates was measured after 6 days of incubation. Each datum point is the mean of at least four measurements of a growing colony. The percentage of growth inhibition was calculated from mean values as follows: Percentage of inhibition = [(mycelial growth under control conditions − mycelial growth in the presence of WSE) /mycelial growth under control conditions] × 100.

### 2.6. Micro-Titer Plate Assay

Chickpea sourdough (CS-T24) WSE was fractionated by ultra-filtration (Ultrafree-MC centrifugal filter units, Millipore, Burlington, MA, USA) using membrane sizes of 30 and 10 kDa cut-off, following the manufacturer’s instructions.

To assess the antifungal activity of WSE and the fractions (WSE_30_, permeate <30 kDa; WSE_10,_ permeate <10 kDa) the micro-titer plate assay, as previously proposed by Muhialdin et al. [40], was used, with some modifications [18]. After growth for 7 days on PDA plates, conidia of *P. roqueforti* DPPMAF1, used as an indicator, were harvested in sterile water containing 0.05% (*v/v*) Tween 80. Counts of the conidia in the suspension were carried out using a Petroff-Hausser counting chamber. Conidia were then resuspended in Malt Extract Broth (MEB, Oxoid Laboratories, Hampshire, United Kingdom) at the density of 10^4^ conidia/mL and placed in 96-well plates. Each well was also complemented with 30% (*v/v*) of the samples. MEB alone (CT1) and inoculated MEB (CT2) added with sterile water (30% *v/v*) instead of samples, were included in the analysis and used as controls. The plates were incubated at 25 °C for 48 h. Fungal growth inhibition was determined by measuring the absorbance at 560 nm through an ELISA reader (Labomed, model UVD-2950, Fremont, CA, USA) and expressed as % of inhibition compared to CT2 (growth without purified fractions).

### 2.7. Proteolysis and Heat Stability of Antifungal Compounds

WSE_10_ was treated with trypsin (EC 3.4.21.4; Sigma Aldrich Co., St. Louis, MO, USA), as described by Atanassova et al. [41]. Trypsin was dissolved in 0.25 M Tris HCl, pH 8 (1%, w/vol, final concentration). The supernatant and the buffered enzyme solution were mixed at a 1:1 ratio. After 5 h of incubation at 25 °C, the reaction was stopped by boiling the mixture for 3 min. After treatment, the pH of the enzymatic treated-WSE (WSEet) was adjusted to 6.0 and the residual activity was determined by the micro-titer plate assay, as described before. The heat stability of the WSE was assessed after heating for 5 min at 100 °C. Afterward, the residual activity of the thermal-treated WSE (WSEtt) was determined by the micro-titer plate assay, as described before.

### 2.8. Purification and Identification of Antifungal Compounds

An aliquot of the partially purified fraction WSE_10_ (corresponding to 10 mg of peptides) was further automatically fractionated (32 fractions for each run) by Reversed-Phase Fast Performance Liquid Chromatography (RP-FPLC), using a Resource RPC column and ÄKTA FPLC equipment, with the UV detector operating at 214 nm (GE Healthcare Bio-Sciences AB, Uppsala, Sweden). Fractions were separated as described in Rizzello et al. [42]. Solvents were removed from collected fractions by freeze-drying. Fractions were re-dissolved in 600 µL sterile water and assayed for the antifungal activity (micro-titer plate assay).

The peptide concentration in the extracts and purified fractions was determined by the *o*-phthaldialdehyde (OPA) method [35].

Purified fractions displaying the highest antifungal activity on the indicator mold were subjected to nano-Liquid Chromatography-Electrospray Ionisation-Mass Spectra/Mass Spectra (nano-LC-ESI-MS/MS), with the aim of identifying peptides. A Finnigan LCQ Deca XP Max ion trap mass spectrometer (ThermoElectron, Waltham, MA, USA) was used through the nano-ESI interface. According to the manufacturer’s instrument settings for nano-LC-ESI-MSMS analyses, MS spectra were automatically recorded by Xcalibur software (ThermoElectron), in positive ion mode. MS/MS spectra were processed using the BioWorks 3.2 software (ThermoElectron), which generated peak lists suitable for database searches. Peptides were identified using the MS/MS ion search of the Mascot search engine (Matrix Science, London, England) and NCBInr protein database (National Centre for Biotechnology Information, Bethesda, MD, USA). For the identification of peptides, the following parameters were considered: Enzyme: “none”; instrument type: “ESI-trap”; peptide mass tolerance: ±0.1%; and fragment mass tolerance: ± 0.5 Da. Results from peptide identification were subjected to a manual evaluation [18], and the validated peptide sequences explained all of the major peaks in the MS/MS spectrum.

### 2.9. Pasta Making and Shelf-Life Investigation

To investigate the effect of the chickpea sourdough (CS-T24) fortification on the microbial shelf-life of the fresh pasta, three types of experimental products were made: A semolina fresh pasta including chickpea sourdough (CS-p); a conventional semolina fresh pasta (CT-p) to be used as the control; and a semolina fresh pasta added of 0.3% (w/w on a flour weight basis) calcium propionate (cP-p). Table 1 summarizes the dough formula used for pasta making. The proximate composition of wheat semolina was as follows: Moisture, 10.0%; protein, 14.5% of dry matter (d.m.); fat, 1.9% of d.m.; available carbohydrates, 78.0% of d.m., dietary fiber 4.2% of d.m.; and ash, 1.3% of. d.m.

Pasta was manufactured using a pilot plant “La parmigiana SG30” (Fidenza, Italy). The doughs had a final DY of 140, corresponding to 29% (w/w) water and 71% (w/w) dry matter (semolina and chickpea flour). CS-T24 was obtained as described above and used as ingredients for pasta making. Sourdough was mixed with durum wheat semolina and water to obtain pasta samples (CS-p). The amount of CS-T24 added to pasta was 10% (w/w on a flour weight basis), which corresponded to the concentration of active molecules contained in the substrate (added with 30% *v/v* WSE) used for the in vitro assays. Ingredients were mixed in three steps (1 min mixing and 6 min hydration). Then, the final dough was mixed for 30 s and extruded at 45–50 °C, through an.76 bronze die (150 mm diameter). The extruded material was cut with a rotating knife for short pasta shapes to obtain grooved “macaroni”.

### 2.10. Nutritional Characterization

The protein (total nitrogen × 5.7), lipids, ash, total dietary fibers (TDF), and moisture contents were determined according to the AACC approved methods 46–11A, 30–10.01, 08–01, 32–05.01, and 44–15A, respectively [28]. The pH and TTA were determined as described before, after homogenization of the pasta in distilled water.

The in vitro protein digestibility (IVPD) and starch hydrolysis (HI) were determined in pasta samples at the optimal cooking time (OCT). IVPD was determined by the method proposed by Akeson and Stahmann [43], with some modifications [44]. Samples were subjected to a sequential enzyme treatment mimicking in vivo digestion in the gastrointestinal tract and IVPD was expressed as the percentage of total protein which was solubilized after enzyme hydrolysis. The evaluation of the starch hydrolysis rate was performed using a procedure mimicking the in vivo digestion of starch [45]. Wheat flour bread (WB) was used as the control to estimate the hydrolysis index (HI = 100). The predicted GI of all pasta samples was calculated using the following equation: pGI = 0.549 × HI + 39.71 [46].

### 2.11. Optimal Cooking Time, Cooking Loss, and Water Absorption

The OCT, corresponding to the disappearance of the white core, was determined as proposed by Schoenlecher et al. [47], and the pH of the cooked fresh pasta was determined as described before. Cooking loss (expressed as grams of matter loss/100 g of pasta) was evaluated by determining the solids lost into the cooking water, as proposed by D’egidio et al. [48]. The increase in pasta weight during cooking (water absorption) was evaluated by weighing the pasta before and after cooking. The results were expressed as [(W1 − W0/W0]/100, where W1 is the weight of cooked pasta and W0 is the weight of the uncooked samples.

### 2.12. Microbiological Shelf-Life

#### 2.12.1. Microbiological and Chemical Analysis during Refrigerated Storage

With the aim of investigating the microbiological shelf-life of the fresh pasta under refrigerated conditions, CS-p, CT-p, and cP-p were stored at 4 °C for 40 days. Specifically, for each type, 15 aliquots of ca. 100 g of pasta were singly packed in polyethylene bags to maintain constant moisture. Three packets of each type of pasta were analyzed during storage (every 10 days), with the aim of enumerating LAB, yeasts, molds, and *Enterobacteriaceae*. The LAB density was determined as described above. Yeasts were plated on Sabouraud Dextrose Agar (SDA, Oxoid), supplemented with chloramphenicol (0.1 g/L) at 25 °C for 48 h. Molds were enumerated on PDA (Oxoid) at 25 °C for 48 h. Total enterobacteria were determined on Violet Red Bile Glucose Agar (VRBGA, Oxoid) at 37 °C for 24 h. The moisture and pH were determined as described before.

#### 2.12.2. Artificial Fungal Contamination and Antifungal Activity in Pasta

For each type, 15 aliquots of ca. 100 g of pasta were inoculated by nebulization with a suspension of 10^2^ conidia/mL of *P. roqueforti* DPPMAF1 [38] and singly packed in polyethylene bags to maintain constant moisture. Packets of the inoculated pasta were incubated at 4 °C for 40 days. The fungal contamination was quantified by a visual observation of the pasta, as a percentage of the macaroni pieces with visible fungal colonies in relation to the total of the packet. Three packets for each type of pasta were analyzed at 10-day intervals.

### 2.13. Statistical Analysis

All analysis, as well as the fermentation, pasta making, and antifungal assays, were carried out in triplicate. The one-way ANOVA, using Tukey’s procedure at *p* < 0.05, was performed for data elaboration (Statistica 12.5, StatSoft Inc., Tulsa, OK, USA).

## 3. Results

### 3.1. Chickpea Sourdough Fermentation

The chickpea flour resulting from the milling of the whole grains was characterized by 9.5 ± 0.4% moisture, 22.0 ± 0.4% (of dry matter, d.m.) proteins, 3.3 ± 0.2% (of d.m.) fat; 49.0 ± 0.5% (of d.m.) carbohydrates, 23.5 ± 0.3% (of d.m.) TDF, and 3.0 ± 0.5% (of d.m.) ash. After the mixing, chickpea dough (CS-T0) had a pH of 6.2 ± 0.2 (Table 2). During the 24 h of incubation required to obtain the CS, the pH decreased by ca. 2 units, and a growth of ca. 2.5 log10 cycles was found in all of the matrices (final LAB cell density in CS-T24 was 9.78 ± 0.11 log10 cfu/g). Significant organic acids synthesis was observed (Table 2) as a consequence of the lactic acid fermentation of the mixed starters, with lactic acid reaching 180 mmol/kg. The FQ was ca. 4.3. Intense proteolysis was observed; indeed, the peptide and TFAA concentration increased, during fermentation, by ca. 3.5- and 2-times, respectively (Table 2). Fermentation also led to degradation of the antinutritional factors (ANF): Phytic acid, raffinose, and condensed tannins decreased 95, 68, and 50%, respectively (Table 2).

### 3.2. Antifungal Activity of the Water/Salt-Soluble Extract from Chickpea Sourdough

**Figure 1:**
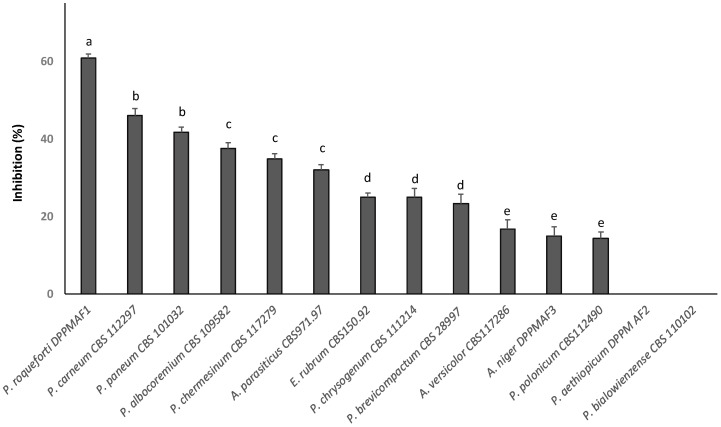
Inhibitory spectrum of the water/salt-soluble extract from chickpea sourdough (30%, *v/v*, on Potato Dextrose Agar (PDA) medium), as determined by the hyphal radial growth rate of fungi, after 6 days of incubation at 25 °C. ^a^^–e^ Values with different superscript letters differ significantly (*p* < 0.05). Each datum point is the mean of at least four measurements of a growing colony. The percentage of growth inhibition was calculated from mean values as follows: % inhibition = [(mycelial growth under control conditions − mycelial growth in the presence of water/salt-soluble extract)/mycelial growth under control conditions] × 100.

The spectrum of activity of the CS-T24 WSE was characterized through the hyphal radial growth rate (Figure 1). Among the 14 species of fungi assessed, only *P. aethiopicum* DPPMAF2 and *P. bialowiezense* CBS110102 growth was not affected by the CS-T24 extract. Among the others, *P. polonicum* CBS 112490, *A. niger* DPPMAF3, and *A. versicolor* CBS117286 were inhibited by less than 20%, while the inhibition of *P. roqueforti* DPPMAF1, *P. paneum* CBS 101032, and *P. carneum* CBS112297 was up to 60% (Figure 1). The WSE obtained from the CS-T0 showed weak antifungal activity (growth inhibition lower than 15%) towards *P. roqueforti* DPPMAF1, *P. paneum* CBS 101032, and *P. carneum* CBS112297.

**Figure 2:**
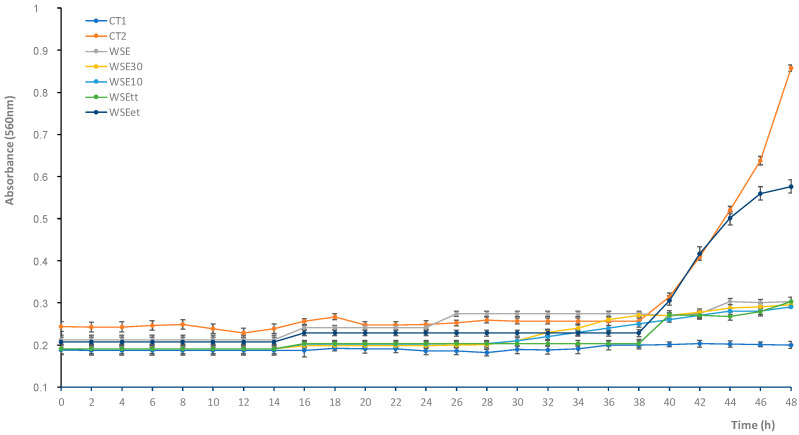
Inhibitory activity of the water/salt-soluble extract (WSE) obtained from chickpea sourdough fermented at 30 °C for 24 h with *Lactoplantibacillus plantarum* LB1 and *Furfurilactobacillus rossiae* LB5. CT1, non-inoculated Malt Extract Broth (MEB) added with 30% (*v/v*) of sterile water; CT2, MEB inoculated with 10^4^ conidia/mL and added with 30% (*v/v*) of sterile water; WSE30 and WSE10: MEB inoculated with 10^4^ conidia/mL and added with 30% (*v/v*) of the ultra-filtration permeates obtained at the cut-off of 30 and 10 KDa, respectively; WSEet and WSEtt: MEB inoculated with 10^4^ conidia/mL and added with 30% (*v/v*) of the WSE subjected to enzymatic or thermal treatment, respectively. Incubation was carried out at 25 °C.

The WSE of CS-T24 was fractionated by ultra-filtration. The antifungal activity, as determined by the micro-titer assay, was found in both of the permeates characterized by molecular masses <30 and <10 kDa (Figure 2). The antifungal activity of both the permeates (ca. 85%) did not significantly (*p* > 0.05) differ from that of the unfractionated WSE. Based on these results, it was hypothesized that active compounds had molecular masses lower than 10 kDa.

The WSE was subjected to enzymatic digestion, in order to verify the potential protein nature of the compounds involved in the antifungal activity. The inhibitory activity of the WSE markedly decreased (−66%) after treatment with trypsin (Figure 2), while after heating, the inhibitory activity was completely retained. This outcome supported the hypothesis of the prevalent peptide-type nature of the antifungal compounds (partial sensitivity to enzymatic digestion and stability to thermal treatment).

### 3.3. Purification and Identification of the Antifungal Compounds

CS-T24-WSE contained 1.9 ± 0.3 mg/mL of peptides. Nevertheless, the ultra-filtration led to an increase of the concentration in the permeate, which was 7.5 ± 0.3 mg/mL in WSE_10_. This partially purified and active fraction was further fractionated by reversed-phase liquid chromatography in thirty-two fractions, five of which (A–E) exhibited inhibitory activity on the *P. roqueforti* DPPMAF1. Active fractions were characterized by a peptide concentration ranging from 3.50 ± 0.07 to 4.22 ± 0.10 mg/mL.

The highest inhibitory activity, as determined by the micro-titer assay, was observed for fractions B and C (67 and 75%, respectively), eluted at 12 and 14% of the acetonitrile gradient, while fractions D and E (Figure S1) (eluted at 17 and 22% of the acetonitrile gradient) displayed activity of 30 and 55%, respectively. A, eluted at 5% of the acetonitrile gradient, exhibited the lowest activity (7%) and it was not subjected to further analysis.

Peptides of the most active fractions (B–E) were identified by nano-LC–ESI–MS-MS, followed by MS-MS and an ion search with the Mascot search engine (Table 3).

A mixture of peptides was identified in all of the extract fractions. The identified peptides, having 12–20 amino acid residues, were characterized by a molecular mass ranging from 1133.6 (ISSATSAIADKA) to 2017.0 (NLESTEQGKGGSDVLGAVKE) Da and a hydrophobic ratio between 25 and 50%. Among these, four were neutral, one (VKSTTTACCDSCVCTK) had a positive net charge, and five had a negative net charge (Table 3).

### 3.4. Assessment of the Pasta Quality and Microbiological Shelf-Life

#### 3.4.1. Nutritional and Technological Features

The fresh pasta samples were characterized by moisture of ca. 28%. No significant (*p* > 0.05) differences were found between CT-p and cP-p (the latter was added with calcium propionate) for any of the nutritional parameters considered. As expected, the pH of the CS-p was significantly (*p* < 0.05) lower than that of the CT-p. Moreover, the activity water was significantly lower in the fortified pasta compared to the reference semolina sample (Table 4).

Compared to this latter sample (Table 4), the main differences in the proximate composition caused by the addition of CS-T24 were the significant (*p* < 0.05) increase of dietary fibers (+46%) and proteins (+9%), and the slight but significant (*p* < 0.05) decrease of carbohydrates (−4%). The CS-T24 also caused a relevant decrease of the HI (−25%). As the consequence, the pGI of the CS-p was 67.49 ± 1.44. Sourdough fermented chickpea also caused an increase of the pasta IVPD, which was ca. 37% higher than that of the semolina pasta samples (Table 4).

The OCT of the CS-p was ca. 1 min shorter than CT-p. Cooking led to a water absorption slightly but significantly (*p* < 0.05) higher in CS-p compared to the semolina samples, while its solid loss was the highest and ca. 17% higher than the control (Table 5). As expected, a marked increase of the pH was observed for cooked CS-p compared to the same uncooked sample (6.0 vs. 5.1).

#### 3.4.2. Microbial Dynamics under Refrigerated Conditions

During the 40-day storage period, a slight but significant (*p* < 0.05) decrease of the moisture was observed. The drop was constant and a final value ranging from 26.2 to 26.3% (Table 6) was observed at the 40th day for all of the fresh pasta samples.

Progressive and slow acidification was observed in CS-p until the 20th day of storage (−0.5 pH unit); then, the value remained almost constant until the 40th day.

During the first 10 days of storage, a relevant pH drop was observed in CT-p and a further but moderate decrease was observed until the 20th day. Even in this case, the value was stable until the end of the period considered. Compared to CT-p, a more gradual decrease of the pH was observed in CP-p in the first 20 days of storage; nevertheless, no significant (*p* < 0.05) differences were found in the final value (Table 6), which corresponded to 5.7–5.8.

**Figure 3:**
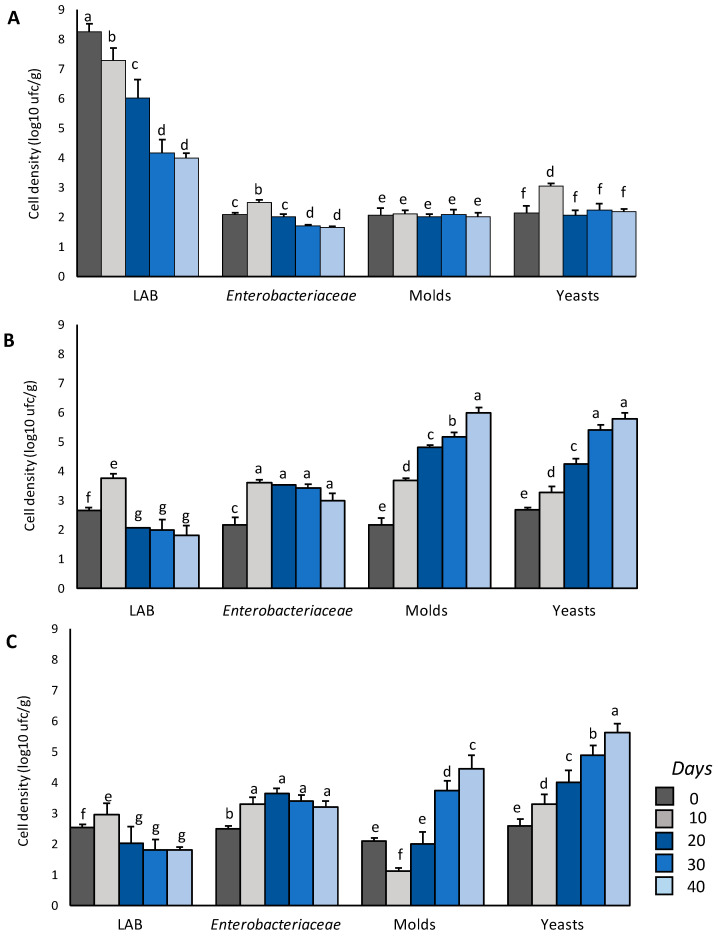
Microbiological analysis of the fresh pasta during a 40-day storage period at 4 °C. Panel (**A**): Semolina fresh pasta fortified with the chickpea sourdough, CS-p; panel (**B**): Conventional semolina fresh pasta CT-p; panel (**C**): Semolina fresh pasta added of 0.3% (w/w on a flour weight basis) calcium propionate, cP-p. LAB, lactic acid bacteria. ^a^^–g^ Values (within the same microbial group and among all the pasta samples) with different superscript letters differ significantly (*p* < 0.05). The data are the means of three independent experiments ± standard deviations (*n* = 3).

As expected, the LAB density was the highest in CS-p, in which ca. 8.26 ± 0.25 log 10 ufc/g was found at the beginning of the storage period (Figure 3). A marked decrease in LAB viability was observed during the first 20 days (ca. 1 log cycle every 10 days). In the last 20 days, the LAB cell density remained stable (ca. 4 log10 ufc/g) (Figure 3). The initial LAB density in CT-p and cP-p was similar (2.55–2.65 log10 cfu/g) and a slight but significant (*p* < 0.05) increase was observed in both samples after the first 10 days of storage. Nevertheless, from then to the end of the storage period, the density decreased and was stable (ca. 2 log10 ufc/g) until the 40th day. The *Enterobacteriaceae* densities were always lower than 2.50 ± 0.10 log10 cfu/g in CS-p, while in CT-p and cP-p, densities in the range of 3–4 log10 ufc/g were observed from the 10th day of storage (Table 6). No mold increase was observed in CS-p for the entire storage period, while significant (*p* < 0.05) and constant increases (ca. 1 cycle every 10 days) were observed in CT-p. Calcium propionate was effective in controlling mold growth until the 30th day; then, the growth increased up to 4.44 ± 0.20 log10 cfu/g. Yeasts in CS-p were always below 2.25 ± 0.16 log10 cfu/g (with the exception of the 10th day), while a progressive increase was observed in CT-p and cP-p (5.80 ± 0.57 and 5.62 ± 0.44 log10 cfu/g were respectively observed at the 40th day of storage) (Figure 3).

#### 3.4.3. Mold Growth Monitoring

The fresh pasta samples were artificially inoculated with *P. roqueforti* conidia and packed in plastic bags to avoid a moisture decrease (potentially affecting the mold growth). The moisture of the samples did not significantly (*p* < 0.05) differ from that reported for non-inoculated pasta (Table 6). The mold-free period under the study conditions was 20 days for CS-p and cP-p and less than 10 days for CT-p (Table 6). Nevertheless, the entity of contamination in CS-p after the first 20 days was markedly smaller compared to cP-p (Table 6).

## 4. Discussion

The short shelf-life of fresh pasta leads to high wastage, generated during the whole production chain, from the industry to the final consumer, and might also be a potential source of food poisoning [49]. With the aim of meeting consumers’ requirements in terms of natural and “clean label” food products and extending the shelf-life of the products, the industry of fresh pasta has been significantly revised [50]. Indeed, research on antifungal compounds, with a focus on plant-derived extracts, essential oils, and those originating during fermentation by microorganisms such as LAB and yeasts, has been carried out in the last 15 years [18,51,52].

Moreover, the growing consumer request for foods with a well-balanced nutritional profile and functional properties has encouraged research on innovation in pasta making. As a staple food, pasta can be considered as a carrier of dietary fiber, vegetable proteins, vitamins, minerals, and functional compounds [24].

The use of fermented ingredients for pasta fortification has recently been investigated under several aspects, including fortification in vitamin B, the reduction of starch digestibility, and the gluten content. LAB fermentation, inspired by sourdough biotechnology, has also been successfully applied to non-conventional flours (e.g., pseudocereals and legumes), in which an overall improvement of the nutritional value, increase in the content of the health-promoting compounds, and significant decrease of ANF were observed [52,53,54,55]. Fermented non-conventional flours, obtained through spontaneous fermentation or by using selected starters, have been proposed as pasta ingredients [24]. Fermentation has also been recognized as an efficient tool for improving the sensory and technological features of fortified pasta. Nevertheless, only a few pasta recipes including the use of fermented ingredients are currently available on the market.

To the best of our knowledge, the use of fermented legume flour as a pasta ingredient for prolonging its microbiological shelf-life has never been investigated.

In the present study, the chickpea flour was fermented using a mixed starter including *L. plantarum* LB1 and *Fr. rossiae* LB5, which were previously successfully used for the fermentation of the wheat germ, conferring an antifungal potential to the matrix due to both the synthesis of organic acids (mainly formic and acetic) and the release, through proteolysis, of antifungal peptides encrypted into native wheat proteins [42]. The chickpea sourdough presented acidification and an organic acids concentration similar to those previously reported in similar fermentation conditions [27]; also, the proteolytic activity was very intense, as expected, since a very high concentration of free amino acids (higher than 2 g/kg) was found at the end of fermentation. Moreover, as largely reported by the literature [52,53,54,55], significant degradation of the ANF, up to 95, 68, and 50% for phytic acid, raffinose, and condensed tannins, respectively, was achieved.

The antifungal activity of the chickpea sourdough extract was observed for a broad spectrum of molds, including *P. roqueforti* DPPMAF1, *P. paneum* CBS 101032, *P. carneum* CBS112297, *E. rubrum* CBS150.92, *P. chrysogenum* CBS 111214, and *P. brevicompactum* CBS28997. Among these, species belonging to the *Penicillium* genus are the most frequently isolated in fresh pasta [56].

Although the antifungal activity of fermented substrates mainly relies on the organic acids synthesized by lactic acid bacteria, these do not fully justify its strength in LAB fermented matrices [23,51]. Hence, the identification of antifungal peptides released through proteolysis during fermentation is required to draw proper conclusions.

The purification procedure employed in this study confirmed that molecules characterized by a mass lower than 10 kDa were responsible for the antifungal activity. Enzymatic treatment of the active water/salt-soluble extract showed a relevant decrease in the activity, thus indicating the proteinaceous nature of the active compounds. The digestion-resistant activity can be ascribed to the organic acids fraction [57,58]. Purification of the extract obtained from the chickpea sourdough through sequential steps, including ultra-filtration and FP- and nano-LC, allowed the identification of a mixture of potentially active peptides. According to the literature [23], the antimicrobial activity of protein hydrolysates is commonly due to the synergistic action of different peptides.

Overall, plant proteins and peptides have been shown to play a pivotal role in in situ fungal resistance [59]. Indeed, antifungal activity was found in the water-soluble extract of *Phaseolus vulgaris* cv. Pinto [37], *Pisum sativum* hydrolysate [60], and a mixture of legume flour (pea, lentil, and fava bean) [61]. Moreover, several proteins, including a glucanase; a chitinase; an antifungal cyclophyllin-like protein [62]; and three antifungal peptides designated cicerin, arietin, and cicearin; were also isolated from the chickpea as part of the natural defense system of the seed towards phytopathogenic fungi [62,63]. However, their effectiveness towards the molds commonly responsible for food spoilage was not previously investigated. Indeed, under the conditions of this work, the antifungal activity of the extract obtained from the unfermented chickpea flour was lower than 15% and it was only observed for three of the spoilage molds included in the study. Moreover, according to the literature, peptides responsible for the antifungal activity in vegetable matrices fermented with LAB are not necessarily released from already active sequences, but acquire the inhibitory activity as a consequence of the hydrolysis [23].

The release of the antifungal peptides is strictly related to the peptidase portfolio of the LAB strains used as starters for fermentation [23]. Therefore, the antifungal effect appears to be specifically related to the starters, the matrix (native proteins and susceptibility to proteolysis), and the degree of proteolysis [23,51].

Despite structural and sequence diversity, antifungal peptides present similar features, such as a small size and the presence of cationic and hydrophobic residues [64,65]. All peptides identified from chickpea sourdough produced in this study resulted in sequences encrypted in *Cicer arietinum* proteins. All had a hydrophobic ratio higher than 25%, due to the presence of alanine (Ala, A), valine (Val, V), leucine (Leu, L), isoleucine (Ile, I), proline (Pro, P), phenylalanine (Phe, F), and cysteine (Cys). Minimal hydrophobicity is required for the penetration and binding of peptides into the bilayer of the cell membrane [64]. VKSTTTACCDSCVCTK was the only cationic peptide with a net charge of +1, an experimental mass of 1876.8 Da, and a total hydrophobic charge of 43%. A positive net charge is generally associated with a strong interaction with the negatively charged cell membrane of microbes [64,66]. In this peptide, Cys residues accounted for circa 25% of the total composition and according to the Antimicrobial Peptide Calculator and Predictor-APD3 [67], it might form (i) a disulfide-bond linked defensin-like beta structure; (ii) helical structures containing S-S bond(s); or (iii) multiple thioether bonds, as in lantibiotics, if the threonine (Thr)/serine (Ser) content is high. Indeed, Thr and Ser accounted for circa 25 and 12%, respectively. Moreover, VKSTTTACCDSCVCTK was encrypted in Bowman-Birk-type proteinase inhibitor-like, which has already been reported to exert antifungal activity [68,69].

Most of the neutral (three out of four) and negative peptides had a high content of glutamate (Glu, up to 15%), arginine (Arg, up to 6%), and glycine (Gly, up to 20%), which were identified in plant-derived peptides with high antifungal activity [70]. All peptides showed 3-5 total hydrophobic residues on the same surface, so the peptides may form α-helix. Linear peptides forming α-helix in a hydrophobic environment may interact with membranes and have the chance to be an antimicrobial peptide [65]. Moreover, the formation of α-helix is considered to be one of the specific structural parameters used to group antifungal peptides [71].

A molecular understanding of the mechanism of action of antifungal peptides is still lacking, although the ability to form membrane pores or alter cytoplasmic membrane septum formation, such as the inhibition of cell-wall/nucleic acid/enzyme synthesis, has been hypothesized [23].

Chickpea sourdough was used as an ingredient for making fresh pasta. Although identified as a promising strategy for the nutritional improvement of conventional semolina pasta, the effects of the fermented legume flours on the main features of the product are only partially known and have been poorly applied to large-scale production [23,27]. Indeed, the characterization of the experimental pasta highlighted some technological differences (a slight decrease of the cooking time and an increase of the cooking loss), but the overall texture and structure were, as previously described, very good. The fortified pasta also exhibited a significant increase in proteins and dietary fiber content, compared to the fresh semolina pasta used as the reference. Raw chickpea has already been considered as an ingredient to fortify semolina pasta with significant nutritional improvements [72]. However, when the chickpea was fermented first, fortified pasta was also characterized by lower concentrations of ANF and improved sensory profiles [24,27].

According to EC Regulation No 1924/2006 [73] on nutrition and health claims on food products, fortified pasta can be labeled as a “source of fiber” and “source of protein”. Indeed, it contains more than 3 g of fiber per 100 g, and proteins account for circa 16% of the total energy of the product.

Compared to the fresh semolina pasta used as the control, a significant increase of the protein digestibility was also observed in the fortified pasta (IVPD of ca. 89%). As previously reported, such an increase mainly depends on the proteolytic activity of the selected starters during fermentation, but also on the activity of the endogenous proteases during the long-term fermentation and the degradation of the antinutritional factors (e.g., phytic acid, condensed tannins, etc.) able to form complexes with proteins, decreasing the possibility of them being degraded by digestive enzymes [52]. LAB have been shown to contribute, through direct and indirect activities, to the decrease of the phytic acid concentration (combined effect of the endogenous phytases activated through the acidification occurring during fermentation and the microbial activity), degradation of the condensed tannins (through several enzymatic activities such as tannase, polyphenol oxidase, and decarboxylase), and hydrolysis of the raffinose through α-galactosidase activity [55,74,75].

Moreover, fortified pasta was also characterized by a relevant decrease of the glycemic index, and thus depended on the biological acidification and the increase in resistant starch (starch fraction not susceptible to the enzymatic digestion) related to the use of the fermented chickpea [52]. The fresh pasta containing the chickpea sourdough was characterized by a starch hydrolysis index of 50.61 (pGI of ca. 67.50).

During storage under refrigerated conditions, fortified pasta, characterized by a pH significantly lower than that of reference pasta, was hostile to the growth of contaminant bacteria and yeasts. Besides the pH, the high cell density of LAB, which remained viable at a high level during refrigerated storage, contributed to the growth limitation of the other microbial groups. Under these conditions of spontaneous contamination, the mold growth in chickpea sourdough fortified pasta was comparable to that observed in pasta added to calcium propionate. To assess the persistence of the antifungal activity, fresh pasta was subjected to an artificial inoculation of *P. roqueforti* and monitored for a 40-day storage period. Under these conditions, pasta fortified with the chickpea sourdough showed a longer shelf-life than pasta added to calcium propionate.

## 5. Conclusions

The present study successfully combined a potential antifungal ingredient (chickpea) and fermentation with selected LAB to extend the shelf-life of fresh pasta. Besides the high content of dietary fibers (4.37%) and proteins (11.20%), nutritional improvements, such as a decrease of ANF and HI (25% lower than the control) and increase of IVPD (36% higher than the control), were achieved in fresh pasta. Moreover, the proteolysis operated form both endogenous proteases (activated by the bio-acidification) and the bacterial specific pool of peptidases led to the release of peptides with high antifungal potential, contributing to the extension of the fortified fresh pasta shelf-life compared to a pasta containing a chemical preservative.

## Figures and Tables

**Table 1:** Formulas of fresh pasta: CS-p, pasta containing 10% (w/w) fermented chickpea sourdough; CT-p, semolina fresh pasta; and cP-p, semolina fresh pasta added of 0.3% (w/w on flour weight basis) calcium propionate.

	Semolina (%)	Water (%)	Chickpea Sourdough		Calcium Propionate (%)
			Chickpea Flour (%)	Water (%)	
CS-p	63.9	24.7	7.1	4.3	
CT-p	71.0	29.0			
cP-p	70.7	29.0			0.3

**Table 2:** Main characteristics of the chickpea sourdough before (CS-T0) and after fermentation (at 30 °C for 24 h, CS-T24) with *Lactoplantibacillus plantarum* LB1 and *Furfurilactobacillus rossiae* LB5.

	CS-T0	CS-T24
Lactic acid bacteria (log10 cfu/g)	7.28 ± 0.24 ^b^	9.78 ± 0.11 ^a^
pH	6.2 ± 0.2 ^a^	4.3 ± 0.1 ^b^
TTA	4.6 ± 0.3 ^b^	28.1 ± 1.2 ^a^
Lactic acid (mmol/kg)	n.d	180.7 ± 4.3
Acetic acid (mmol/kg)	n.d.	42.7 ± 0.7
Fermentation quotient (FQ)	-	4.3 ± 0.2
Peptides (mg/kg)	750 ± 16 ^b^	2600 ± 50 ^a^
TFAA (mg/kg)	965 ± 10 ^b^	2049 ± 15 ^a^
Phytic acid g/100g	2.72 ± 0.05 ^a^	0.13 ± 0.05 ^b^
Raffinose g/kg	1.88 ± 0.09 ^a^	0.60 ± 0.06 ^b^
Condensed tannins g/kg	0.90 ± 0.02 ^a^	0.45 ± 0.05 ^b^

TTA, Total Titratable Acidity (mL NaOH 0.1M/10 g sample); TFAA, Total Free Amino Acids. ^a^^–b^ Values in the same row with different superscript letters differ significantly (*p* < 0.05). The data are the means of three independent experiments ± standard deviations (*n* = 3). n.d., not detected.

**Table 3:** List of peptides identified in partially purified peptide fractions obtained from the water/salt-soluble extract from chickpea sourdough through Reversed-Phase Fast Performance Liquid Chromatography (RP-RPLC).

Fraction	Sequence	Experimental Mass (Da)	Calculated Mass (Da)	Δmass (Da)	Length	Net Charge	Hydrophobic Ratio (%)	Accession Number (Protein)	Fragment
**B**	ISSATSAIADKA	1133.5914	1133.5928	0.0014	12	0	50	XP_004515723.2 (LIM domain-containing protein A-like isoform X1)	645–656
**C**	NLESTEQGKGGSDVLGAVKE	2016.9928	2016.9964	0.0036	20	−2	25	XP_004487170.1 (seed biotin-containing protein SBP65)	559–578
	TGQYADDAAEDTRDFAGE	1930.7765	1930.7817	0.0052	18	−5	27	XP_004508082.1 (embryonic protein DC-8-like)	177–194
	KIQDVATGAGEYTAEK	1679.8348	1679.8366	0.0018	16	−1	31		373–388
	ASNIADAGKDTFEAAK	1607.7778	1607.7791	0.0013	16	−1	43		389–404
**D**	AGESIKETAANVGASAK	1602.8208	1602.8213	0.0005	17	0	41	NP_001266059.1 (18 kDa seed maturation protein-like)	7–23
	ESAGFVGHETATNIAR	1658.8004	1658.8012	0.0008	16	−0.75	37	XP_012567985.1 (late embryogenesis abundant protein D-34-like isoform X1)	67–82
**E**	NVVSSIGETVGK	1188.6354	1188.6350	0.0004	12	0	33	XP_004487170.1 (seed biotin-containing protein SBP65)	491–502
	VKSTTTACCDSCVCTK	1876.7769	1876.7788	0.0019	16	1	43	XP_004492724.1 (Bowman-Birk type proteinase inhibitor-like)	49–64
	SVADVAGYVGQK	1192.6079	1192.6088	0.0009	12	0	41	XP_004489219.1 (P24 oleosin isoform A-like)	156–167

**Table 4:** Main nutritional properties of the fresh pasta: CS-p, fresh pasta fortified with the chickpea sourdough; CT-p, conventional semolina fresh pasta; CT-p, cP-p semolina fresh pasta added to 0.3% (w/w on a flour weight basis) calcium propionate.

	CS-p	CT-p	cP-p
Moisture (%)	28.1 ± 0.27 ^a^	28.5 ± 0.15 ^a^	28.2 ± 0.18 ^a^
pH	5.1 ± 0.2 ^b^	6.5 ± 0.2 ^a^	6.5 ± 0.2 ^a^
TTA	4.8 ± 0.35 ^a^	2.10 ± 0.22 ^b^	2.15 ± 0.17 ^b^
Water activity	0.971 ± 0.002 ^b^	0.975 ± 0.002 ^a^	0.975 ± 0.002 ^a^
Protein (%)	11.2 ± 0.27 ^a^	10.25 ± 0.09 ^b^	10.12 ± 0.08 ^b^
Fat (%)	1.40 ± 0.02 ^a^	1.35 ± 0.10 ^a^	1.33 ± 0.04 ^a^
Available carbohydrates (%)	53.15 ± 0.21 ^b^	55.32 ± 0.17 ^a^	55.33 ± 0.10 ^a^
Total dietary fibers (%)	4.37 ± 0.31 ^a^	2.98 ± 0.08 ^b^	2.88 ± 0.18 ^b^
Ash (%)	1.05 ± 0.05 ^a^	0.92 ± 0.05 ^b^	0.94 ± 0.03 ^b^
HI (%)	50.61 ± 1.23 ^b^	67.5 ± 1.1 ^a^	67.0 ± 1.8 ^a^
pGI	67.49 ± 1.04 ^b^	76.76 ± 0.98 ^a^	74.35 ± 1.98 ^a^
IVPD (%)	88.80 ± 0.50 ^a^	65.1 ± 1.42 ^b^	63.1 ± 2.02 ^b^

TTA, Total Titratable Acidity (mL NaOH 0.1M/10 g sample); HI, Starch Hydrolysis Index; pGI, predicted Glycemic Index; IVPD, in vitro protein digestibility. ^a^^–b^ Values in the same row with different superscript letters differ significantly (*p* < 0.05). The data are the means of three independent experiments ± standard deviations (*n* = 3).

**Table 5:** Main technological properties of the fresh pasta: CS-p, semolina fresh pasta fortified with the chickpea sourdough; CT-p, conventional semolina fresh pasta; CT-p, cP-p semolina fresh pasta added to 0.3% (w/w on a flour weight basis) calcium propionate.

	CS-p	CT-p	cP-p
OCT (min)	3.10 ± 0.20 ^b^	4.25 ± 0.20 ^a^	4.30 ± 0.10 ^a^
Water Absorption (%)	85 ± 2 ^a^	80 ± 2 ^b^	80 ± 2 ^b^
Cooking loss (% of d.m.)	4.57 ± 0.12 ^a^	3.91 ± 0.10 ^b^	3.80 ± 0.15 ^b^
pH (at the OCT)	6.0 ± 0.1 ^b^	6.7 ± 0.2 ^a^	6.7 ± 0.1 ^a^

OCT, Optimal cooking time; d.m., dry matter. ^a^^–b^ Values in the same row with different superscript letters differ significantly (*p* < 0.05). The data are the means of three independent experiments ± standard deviations (*n* = 3).

**Table 6:** Fungal contamination of the fresh pasta inoculated with *Penicillium roqueforti* DPPMAF1 during a 40-day storage period at 4 °C. CS-p, semolina fresh pasta fortified with the chickpea sourdough; CT-p, conventional semolina fresh pasta; CT-p, cP-p semolina fresh pasta added to 0.3% (w/w on a flour weight basis) calcium propionate. The pH and moisture are also reported.

	CS-p					CT-p					cP-p				
**Storage time (days)**	0	10	20	30	40	0	10	20	30	40	0	10	20	30	40
**Moisture**	28.1 ± 0.27 ^a^	27.5 ± 0.07 ^b^	27.1 ± 0.11 ^c^	26.5 ± 0.12 ^d^	26.2 ± 0.07 ^e^	28.5 ± 0.15 ^a^	27.4 ± 0.10 ^b^	27.1 ± 0.08 ^c^	26.6 ± 0.05 ^d^	26.3 ± 0.05 ^e^	28.2 ± 0.18 ^a^	27.5 ± 0.13 ^b^	27.2 ± 0.09 ^c^	26.5 ± 0.10 ^d^	26.2 ± 0.13 ^e^
**pH**	5.1 ± 0.2 ^c^	4.8 ± 0.1 ^d^	4.5 ± 0.1 ^e^	4.4 ± 0.2 ^e^	4.4 ± 0.2 ^e^	6.5 ± 0.2 ^a^	6.0 ± 0.1 ^b^	5.8 ± 0.2 ^b^	5.8 ± 0.2 ^b^	5.7 ± 0.1 ^b^	6.5 ± 0.2 ^a^	6.3 ± 0.2 ^a^	6.2 ± 0.1 ^a^	5.9 ± 0.2 ^b^	5.8 ± 0.2 ^b^
***FC***	−	−	−	+	+	−	++	+++	++++	++++	−	−	−	++	+++

^a^^–e^ Values in the same row with different superscript letters differ significantly (*p* < 0.05). The data are the means of three independent experiments ± standard deviations (*n* = 3). FC, fungal contamination has been estimated as follows: “−“, no fungal growth observed; “+”, colonies present on 1–25% of the macaroni pieces (mean of the percentage of three different packets); “++”, 26–50%; “+++”, 51–75%; and “++++”, 75–100%.

## Supplementary Materials

The following are available online at https://www.mdpi.com/2076-2607/8/9/1322/s1: Figure S1: Representative RP-FPLC chromatogram (UV detector 214 nm) of the fraction WSE_10_ obtained from the chickpea sourdough. The gradient of eluent B is represented by the dashed line. A–E refer to the fractions with antifungal activity.
