# Supplementary material for: Extension of the Shelf-Life of Fresh Pasta Using Chickpea Flour Fermented with Selected Lactic Acid Bacteria
Source: Microorganisms. 2020 Aug 30;8(9):1322. doi: 10.3390/microorganisms8091322 (PMC7564801; doi:10.3390/microorganisms8091322)

Figure S1. Representative RP-FPLC chromatogram (UV detector 214 nm) of the fraction WSE10 obtained from the chickpea sourdough. The gradient of the eluent B was represented by the dashed line. A-E refer to the fractions with antifungal activity.

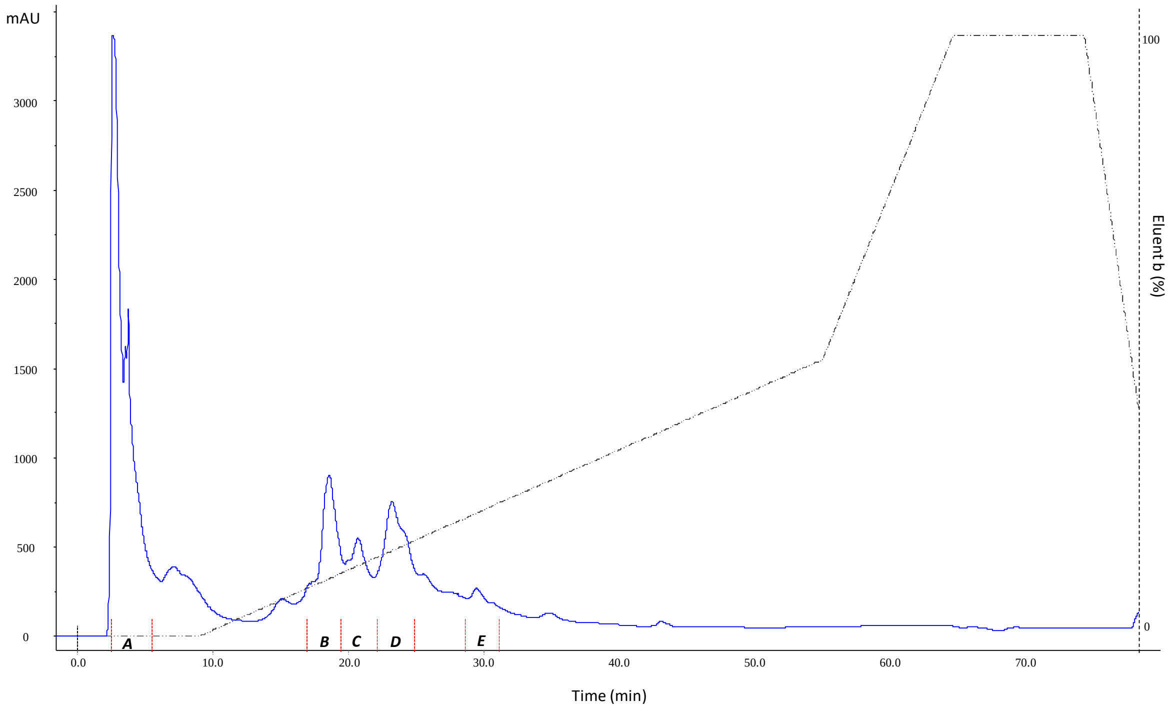

Supplement: Supplementary file 1 [file microorganisms-08-01322-s001.pdf]
